# Acupuncture in Women with Human Polycystic Ovary/Ovarian Syndrome: Protocol for a Randomized Controlled Trial

**DOI:** 10.3390/healthcare10101999

**Published:** 2022-10-11

**Authors:** Natália M. de Oliveira, Jorge Machado, Zaiwei Huang, Maria Begoña Criado

**Affiliations:** 1 ICBAS-Abel Salazar Institute for Biomedical Sciences, University of Porto, 4099-002 Porto, Portugal; 2 CBSin-Center of BioSciences in Integrative Health, 4000-105 Porto, Portugal; 3 LABIOMEP–Porto Biomechanics Laboratory, University of Porto, 4200-450 Porto, Portugal; 4 IEC-International Education College, Zhejiang Chinese Medical University, Hangzhou 310053, China; 5 TOXRUN-CESPU Toxicology Research Unit, University Institute of Health Sciences, CESPU, 4585-116 Gandra, Portugal

**Keywords:** PCOS, metabolic dysfunction, mental health, TCM, acupuncture therapy

## Abstract

(1) Background: Human polycystic ovary/ovarian syndrome (PCOS) is linked to endocrine, metabolic, and psychological complications. We propose a randomized controlled pilot study for an acupuncture protocol regarding the management of PCOS symptoms based on TCM diagnosis; (2) Methods: We will randomly allocate 120 women diagnosed with PCOS into two groups. The study group will be treated with acupuncture for points known to act upon the autonomous regulation of the hormonal, metabolic and emotional components. (3) Results and Conclusions: We expect to provide evidence of high methodological quality related to the effects and safety of an acupuncture protocol based on the perspective of a TCM diagnostic.

## 1. Introduction

Polycystic ovary/ovarian syndrome (PCOS) is considered the most frequent endocrine pathophysiology among women of fertile age, displaying a prevalence between 5 and 15% relative to the clinical diagnostic criteria [1]. Azziz et al., 2004, estimated an annual cost of about 4.37 billion dollars spent in identification and management of the PCOS condition in the USA, which demonstrates the importance of investing in solutions for this condition’s symptoms. With a still unidentified origin, the diagnostic of this syndrome is always made by exclusion of conditions that mimic the phenotypes [2]. In 2003, the European Society for Human Reproduction and Embryology (ESHRE) and the American Society for Reproductive Medicine (ASRM) reformulated the diagnosis criteria of PCOS. Two out of the following three criteria should be present to confirm the syndrome: oligo/anovulation, androgen excess, and the confirmation of polycystic ovarian morphology by imaging. These are designed as Rotterdam criteria, and from them, it is possible to identify four distinct phenotypes of PCOS (Table 1). PCOS has been strongly associated with alteration in the autonomic nervous system: namely, increased sympathetic nervous activity [3]. Endocrine and metabolic implications of the syndrome reflect a high comorbidity [4] within three major aspects: hyperandrogenism [5,6,7,8] and reproductive issues [9,10,11,12], metabolic [13,14,15,16,17,18], and mental or emotional [19,20,21,22,23,24,25,26]. Different clinical strategies have been designed for PCOS management according to international guidelines [11,27,28,29]. However, allopathic therapies have shown several unpleasant secondary effects, which motivate PCOS women to search for complementary medicines such as traditional Chinese medicine (TCM). Among the TCM therapeutics, acupuncture has been used as therapy or co-therapy to improve the comorbidity’s symptoms affecting women with this condition. Diverse studies using low frequency electroacupuncture (EA), alone [30,31] or in combination with exercise to modulate the sympathetic system, concluded that acupuncture seems to regulate the hypersecretion of androgens, improving both the reproductive and endocrine metabolic functional state [32]. Acupuncture has also been used, alone or in combination with clomiphene, to ameliorate the disorder of subfertility in PCOS patients [33,34]. With respect to psychological balance, acupuncture studies were designed to relieve anxiety and depression in PCOS patients [35]. Acupuncture therapy is thought to have a positive impact on the treatment of PCOS through the following mechanisms: sympathetic output modulation through spinal reflexes [36]; modulation of hypothalamic–pituitary–adrenal (HPA) and hypothalamic–pituitary–gonadal (HPG) axes through the increase of β-endorphin secretion [37,38,39,40,41,42]; and influencing insulin sensitivity or resistance through functional balance of the insulin signaling pathways [43]. PCOS in TCM is considered a complex combination of different factors that end up in blockage of the communication between the Penetrating (Chong Mai) and Conception (Ren Mai) vessels, compromising the female’s fertility and reproductive cycle normalization, with or without association to pathogenic heat and phlegm (Lyttleton, 2013). Usually, patients present a tendency to a kidney Yang and/or Yin deficiency, possibly associated to a Qi deficiency in spleen or lung, hence leading to a weakened Shen in the long run. On the other hand, we can have a scenario where kidney Yang deficiency may be associated to Xue stasis leading to cardiac phlegm/dampness in the long run, and, therefore, a disturbed Shen. Main pathogenic factors generating this condition may be stress/overworking, emotional origin (sadness, fear, or excessive worry), cold or phlegm/dampness. According to Maciocia, the kidney is the internal organ most closely associated to the ability to conceive, therefore kidney deficiency is the pattern most commonly found among gynecological pathologies. Kidney deficiency can be due to kidney Yin and/or kidney Yang deficiency. The same author referenced that the overlap of blood and kidney deficiency are quite common due to the relation of kidneys/uterus/blood/Penetrating vessel (Chong Mai). The Penetrating vessel supplies blood to the uterus; however, this supply is also managed by the provision of blood from the liver, the internal organ that stores the blood. So, this liver-blood is key for a healthy menstrual function. Not to mention that, according to the Five Element Theory, Water is the mother of Wood, meaning that the kidney organ is the mother of the liver which is consensual with the relation between essence and blood: kidneys store Jing/essence and the liver stores blood; hence, essence is the mother of the blood. Most published studies of acupuncture in PCOS are concerned with anovulatory cycles and sub/infertility, addressed in TCM as TianGui disorder [44], which is the disorder for which most women seek for help in complementary medicine. However, many women also search for the treatment of symptoms associated with hyperandrogenism either for aesthetic reason, such as acne and alopecia, or for derived comorbidities such as overweight, insulin resistance, diabetes type II, and others. Recent analysis of obstetric outcomes in TCM syndromes showed that possibly more than the usual two distinctions between kidney deficiency and phlegm-dampness and/or blood stasis may be found in women diagnosed with PCOS. Wang et al. (2017) made a secondary analysis of the results of the RCT of Wu et al., 2016, and concluded that it is possible to identify four TCM phenotypes that could be correlated with the four phenotypes according to the Rotterdam criteria (Table 1). According to the author, these four TCM phenotypes would be: “kidney deficiency and liver stasis” (40.29%), “spleen deficiency and phlegm stasis” (32.27%), “phlegm stasis and blood stasis” (16.55%), and “kidney deficiency and blood stasis” (10.89%)—to help in understanding the syndromes according to TCM, we can leave a quick definition of these patterns regarding the reproductive function. According to Lyttleton, kidney deficiency can be translated in symptoms such as in the study, where kidney deficiency with liver stasis patients showed a tendency to have psychological or mental problems [45]. Spleen deficiency and phlegm stasis patients presented metabolic abnormalities, including higher body weights, body mass index, waist and hip circumferences, systolic blood pressure, free androgen index, triglycerides, alanine transaminase, and occurrence of metabolism syndrome. Patients with phlegm and blood stasis had higher free testosterone and insulin, and patients with kidney deficiency with blood stasis presented a more severe reproductive disorder including higher luteinizing hormone and the ratio of luteinizing hormone to follicle stimulation hormone. In general, patterns derived from kidney deficiency demonstrated an association with psychological or mental problems, while patterns associated to phlegm syndrome had more severe glucolipid metabolic abnormalities.

**Figure 1:**
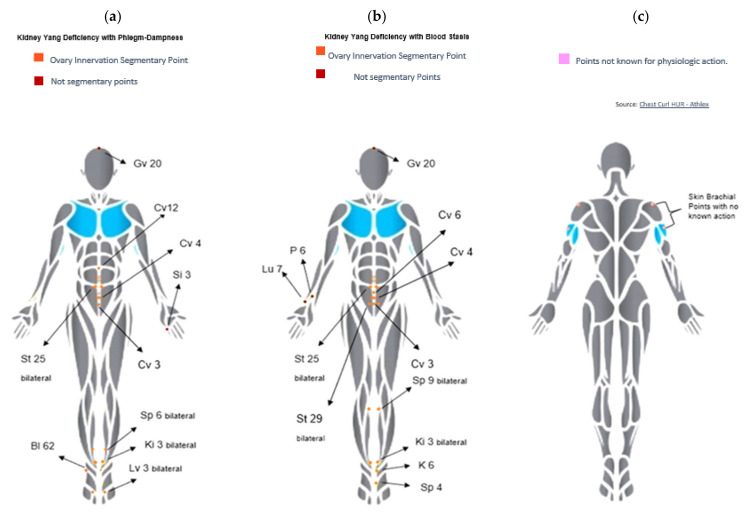
Diagram depiction of the active and sham acupuncture treatments. (**a**) Acupuncture Protocol A; (**b**) Acupuncture Protocol B; (**c**) Sham Acupuncture Protocol.

According to what was mentioned before, it would be an important contribution for PCOS patient treatment to perform an acupuncture randomized controlled trial according to TCM diagnosis of the condition. In this sense, we made a revision of the literature produced during the last five years on TCM PCOS management (data not shown), and we found important limitations that we will try to overcome with the proposed study. So, we designed an acupuncture RCT study according to the TCM pathomechanism associated with PCOS proposed by Lyttleton (2013). According to this proposed mechanism, impairment of the Conception and Governing vessels is a common denominator to gynecological problems. Moreover, Conception and Governing vessel deficiencies are closely related to kidney deficiency. Most women diagnosed with PCOS according to the Rotterdam criteria show Phenotype A or 1 and Phenotype C or 3 (Table 1), which are both characterized by polycystic ovary morphology plus ovulatory disfunction, and in the case of the first phenotype, it is also considered that the diagnosis will show the presence of signs and symptoms of hyperandrogenism. Attending to the common denominator of ovulatory disfunction, we can consider the TCM pattern of kidney deficiency in both main groups, because when the kidney Yin is compromised, the glands and hormonal regulation of the body is considered to be compromised as well—the kidney is the internal organ in TCM associated with the reproductive capacity as well to the development of the fetus. On the other hand, the polycystic ovary morphology can be related to not only kidney deficiency but also to stagnant blood and liver Qi. If liver Qi is stagnant for too long, the function of the body where the liver channel travels might be altered/blocked so we can find pain and difficulty in the egg release in the ovaries as well as the formation of cysts for anovulation. This cyst formation is considered to be the presence of phlegm-dampness in the reproductive system. So, thereby, that is the reasoning behind the kidney deficiency + phlegm-dampness. According to TCM, Qi (energy) is considered the mother of the Xue (blood), meaning that if the flow of qi is compromised in the body so is the flow of Xue. The kidney Yang deficiency in some women will manifest as a deficient qi and, to some degree, it will compromise blood microcirculation (designated as Xue stasis in TCM) affecting the function of the internal organ heart in TCM, leading to Heart Fire. This Heart Fire not only is manifested by anxiety but also causes or worsens the pre-existence of kidney deficiency, especially kidney Yin deficiency. Thus, we justified the simple division in the second branch of patterns: kidney deficiency + blood stasis. Blood stasis might be also concomitant with the presence of blood deficiency (either for blood loss, unhealthy dietetic habits, overwork, or even as ethiological—part of the constitution of that female individual). Normally, when the level of blood is low, also the flow or its circulation is often slower. According to Maciocia, to address the Extraordinary vessels is absolutely essential in the treatment of PCOS for the reason that it will allow to resolve the dampness-phlegm from the uterus and to tonify the kidneys (and, therefore, regulate the hormones). In this way, from tonifying the kidney, we expect a better functioning of the Conception vessel, moving stagnant Qi and contributing to unblocking uterus blood stasis—present in kidney deficiency associated with blood stasis syndrome and to deblock phlegm-dampness, present in kidney deficiency associated with phlegm-dampness syndrome. Thus, for women diagnosed with kidney deficiency associated with phlegm-dampness syndrome and/or with the main symptoms of spleen deficiency or liver stasis, we propose Protocol A of acupuncture (Table A1 and Figure 1a), and for women diagnosed with kidney deficiency with the main symptoms of blood stasis (with or without phlegm stasis), we propose Protocol B (Table A1 and Figure 1b).

## 2. Materials and Methods

### 2.1. Study Design

We propose a randomized, sham-controlled, single-blinded trial that aims to ameliorate PCOS symptoms, namely, clinical and biochemical hyperandrogenism, ovulation rate, glucolipidic profile, IR, metabolic syndrome, and mental health-related parameters such as anxiety, depression, and quality of life in women diagnosed with this condition. The proposed study trial follows the SPIRIT guidelines and checklist.

### 2.2. Recruitment, Participants, and Randomization

**Figure 2:**
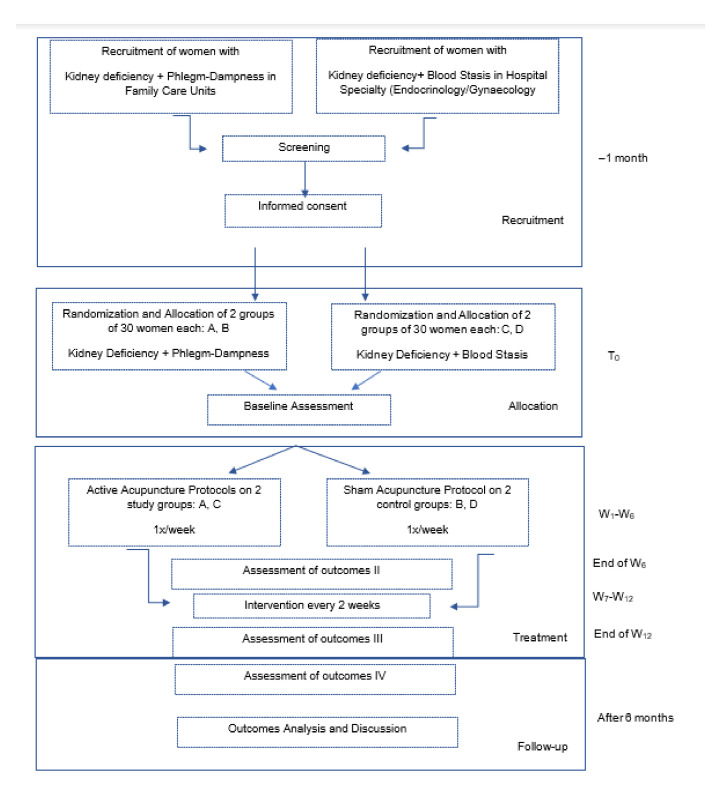
Flowchart of the study period.

We propose a two-armed pilot study comparing the effects of active acupuncture (SG) with sham acupuncture (CG). The flow chart of the study design is shown in Figure 2. The sample will be 120 women, divided in two main groups according to the TCM diagnosis: Group A (*n* = 60): kidney deficiency associated with phlegm-dampness syndrome and/or spleen deficiency or liver stasis and group B (*n* = 60): kidney deficiency with main symptoms of blood stasis. Each group should have a total of 60 participants to achieve statistical difference in the case of 20% withdrawal of the participants [34]. Participants will be recruited directly from the family doctor and/or hospital specialty of gynecology/endocrinology. Each medical practitioner will refer patients with PCOS diagnostic aged between 18 and 59 y.o. with main symptoms of metabolic imbalance and women between 18 and 40 y.o. with main symptoms of hyperandrogenism and fertility issues to the principal investigator to be later recruited by telephone interview. Patients who show interest in participating will first be prescreened and recorded by an intake to verify that they meet basic inclusion criteria. All questions about the study, as well as the written informed consent, will be explained in detail. The goal of the screening visit will be to confirm a diagnosis of PCOS and to exclude major medical illnesses. These tests will aim to confirm the western and TCM diagnostic. Women diagnosed with kidney deficiency associated with phlegm-dampness will be randomly allocated by a computer program in a 1:1 ratio to two groups of 30 individuals each, one will be the study group (SGA) submitted to active acupuncture, and the other will be the control group submitted to sham acupuncture (CGA). The same procedure design will be applied to the 60 women diagnosed with kidney deficiency associated with blood stasis (SGB and CGB). All baseline and follow-up measures should be obtained on a single day. However, questionnaires may be spread between the screening and the baseline visit (Table 2).

### 2.3. Inclusion Criteria

Diagnosis of PCOS according to the Rotterdam criteria (Revised 2003 Consensus on Diagnostic Criteria and Long-Term Health Risks Related to Polycystic Ovary Syndrome, 2004). Hyperandrogenism will be evaluated through hirsutism, alopecia, acne, or through the calculation of total free androgen when clinical signs are not clear [48]. Hirsutism should be assessed using the modified Ferriman–Gallwey score (mFG), where a level ≥ 4 to 6 indicates hirsutism. Alopecia will be assessed using the Ludwig visual score. For acne, no universally accepted visual assessment is available. When clinical signs of hyperandrogenism are unclear or absent, measurement of calculated free testosterone, free androgen index, or calculated bioavailable testosterone should be undertaken [48]. Ovulatory dysfunction is clinically reflected by irregular menstrual cycles, which are defined as less than 21 or more than 35 days in women over 3 years postmenarche to perimenopause.

### 2.4. Exclusion Criteria

The exclusion criteria will be women diagnosed with congenital adrenal hyperplasia (CAH), androgen-secreting tumors, Cushing’s syndrome, thyroid dysfunction, and hyperprolactinemia; women below 18 y.o.; pregnancy within the past 6 weeks; within 6 weeks postabortion or postpartum; breastfeeding within the last 6 months; not willing to give written consent to the study; patients with a history of heart problems, stroke, seizures, or epilepsy.

### 2.5. Intervention

A schedule was established for a total of 12 weeks of intervention, with a frequency of treatment of 1 time per week during the first 6 weeks and 2 times per month in the following 6 weeks. Treatments will take place at a research center/private TCM clinic (Figure 2 and Table 2).

#### 2.5.1. Active Acupuncture

Points: Traditional Chinese medicine regards the PCOS condition as a mass development syndrome such as kidney Yang deficiency and phlegm plus dampness and/or blood stasis. So, treatment should address the resolution of dampness in the lower Triple Burner, eliminate stasis promoting blood circulation, thus leading to an improved Penetrating (Chong Mai) and Conception (Ren Mai) vessel communication, reinvigorate kidney Yang, harmonize Qi circulation in the spleen and lung vessels, and dispel possible internal heat as well as lead to a clear Shen. Chosen points for this study protocol are discriminated and justified in Table A1 and Figure 1a–c.

In short, the combination of Gǔangyúan REN-4 with Tàixī KID-3 and Tàichōng LIV-3, both Earth points, contributes to treat kidney deficiency and tonify liver blood, soothing the liver’s Yang hyperactivity, and rooting the Shen, thus, calming emotions [49]. The sedation of Bàihuì DU-20 may add to the relief of anxiety. On the other hand, Zhōngjí REN-3 and Zhōngwăn REN-12 will also contribute to moving the stagnated Qi associated with phlegm-dampness syndrome. Stimulating points in the lower Triple Burner, namely, Shēnmài BL-62 and Si3 Hòuxī SI-3, we expect an improvement of issues related to food, fluids, blood stasis, accumulated phlegm, and stagnant emotions derived from kidney deficiency associated with phlegm-dampness syndrome. The Qi stagnation observed in these patients usually leads to an imbalance in the transforming function of the Earth element for which Tiānshū ST-25 and Sānyīnjiāo SP-6 may help to restore the Qi and Xue circulation for a general improvement of homeostasis [45]. In women presenting blood stasis, the Conception–Governor vessel circulation is prone to an imbalance that leads to frequent amenorrhea and fertility issues. For that, we suggest regulating the Conception vessel to promote a healthy reproductive cycle by opening Ren Mai and restoring harmony by pairing the Luo points Zhàohăi KID-6 with right Lièquè LU-7 and right Nèiguān P-6 with left Gōngsūn SP-4 to regulate the Conception, Governing, and Penetrating vessels [49]. We would also add Tiānshū ST-25, Guīlái ST-29, and Yīlíngqúan SP-9 to stabilize and harmonize the Earth movement, nourishing blood and eliminate stasis with the spleen’s reinforcement [45]. Based on the characteristics of the referred points, we designed two protocols: Protocol A includes: Zhōngjí REN-3 (dispel), Gǔangyúan REN-4 (tonify), Zhōngwăn REN-12 (dispel), and Tiānshū ST-25 bilaterally (dispel) and Sānyīnjiāo SP-6 bilaterally (tonify). Needles will also be placed in extra segmental acupuncture points: Bàihui DU-20 (sedation), Tàichōng LIV-3 (dispel), Tàixī KID-3 (tonify), Shēnmài BL-62 (dispel), and Hòuxī SI-3 (tonify). In total, 14 needles will be placed (Table A1, and Figure 1a). Protocol B: consists of 16 needles placed in segmental abdominal points; Zhōngjí REN-3 (dispel), Gǔangyúan REN-4 (tonify), Qìhăi REN-6 (tonify), Tiānshū ST-25 (dispel), Guīlái ST-29 (dispel), bilaterally, and extra segmental points: Yīlíngqúan SP-9 bilaterally (tonify), Nèiguān P-6, left Gōngsūn SP-4 (tonify), left Zhàohăi KID-6 (tonify), Tàixī KID-3 (tonify bilaterally), right Lièquè LU-7 (tonify), and Bàihuì DU-20 (sedate) (Table A1 and Figure 1b). Needles and insertion: disposable 25 **×** 25 needle will be used. Insertion depth will be 15–35 mm into skeletal muscle or fibrous tissue. The depth of needle insertion could vary according to the location depth of the acupoint between patients depending on body mass index (BMI), as depth should be enough to reach muscle/fibrous tissue. Stimulation: When needles are inserted, they will be stimulated by manual rotation circa 180 degrees back and forth to evoke tingling sensation—so called de Qi—reflecting activation of sensory afferents. As soon as Qi sensation is reached, the needle should not hurt or cause any pain or discomfort. After 10 min, needles will be stimulated manually. This should be repeated after 20 min and again after 30 min. Two sets of acupuncture points will be used according to the symptoms of each study group.

#### 2.5.2. Sham Acupuncture

Needle insertion: In the control acupuncture protocol, needling (single use of needle per session) should be gentle, superficial, and oblique on each shoulder and upper arm at non-acupuncture points. This needling placement does not correspond to any known acupoints; thus, it is unlikely to affect any segment innervation related to ovaries or implicated in PCOS physiology (Table A1 and Figure 1c). Stimulation: No manual stimulation, whether tonify or disperse, will be done during the intervention.

## 3. Results

The target of this study focuses on four types of parameters divided into primary, secondary, and exploratory outcomes. Primary outcomes refer to hormonal parameters, and secondary to anthropometric and metabolic parameters. Exploratory outcomes are related to quality-of-life analysis and will be performed using the Medical Outcomes Study 36-Item Short Form Health Survey [50], the Zung Self-Rating Anxiety Scale [51], and the Zung Self-Rating Depression Scale [52] (Table 3). All the questionnaires used are validated for Portuguese language. Outcome measures will be collected at baseline, 6 weeks, 12 weeks, and 6 months after the last acupuncture treatment (Table 2).

### 3.1. Blinded

A random code and blind code will be conducted by a “third party” independent of the study using opaque envelopes. The envelopes will be sealed and shuffled, and the assignment records will not be disclosed until the end of the study. Trial participants, formal physicians, therapists, outcome assessors, and data analysts will be blinded to the treatment allocation to minimize potential sources of bias. Only acupuncturists will not be blinded to the treatment allocation. Licensed acupuncturist profile with at least five years of experience will be recruited and will be trained to administer acupuncture. The techniques for the entire treatment procedure will be standardized between practitioners. Results will be kept sealed until the end of the intervention phase. Data analysis will be conducted independently by an experienced statistician, independent of the research team.

### 3.2. Adverse Events/Serious Reactions

Adverse events will be monitored for each treatment during the trial. Any adverse events or reactions that are thought to be causally associated with the intervention will be recorded, managed, and reported to the study coordinators. Serious adverse events or severe adverse reactions will be defined according to the International Harmonization Harmonized Tripartite Guideline and reported to the ethical committee.

### 3.3. Data Collection and Statistical Analysis

All data will be entered into a predesigned, password protected electronic database by two independent researchers who will be blinded to the allocation groups. All statistical analyses of the data will be performed using the SPSS program V.21.0. A *p* value < 0.05 will be considered statistically significant. For cases in which we do not finish all the treatment, we selected intention-to-treat (ITT) analysis to avoid the effects of crossover and drop-out. Measured data will be expressed as mean ± SD (x ±s) if it obeys a normal distribution or approximate normal distribution. The median (interquartile range [IQR]) will be used if the data do not obey the normal distribution and count data will be expressed in terms of the number of cases. We will use chi-square tests for categorical data and two sample *t*-tests or Wilcoxon rank sum tests for continuous data, according to whether the data are normally distributed or not. The variance analysis will be performed for the difference between groups and within the group. The stratified analysis will be performed to control the confounding factor if necessary.

### 3.4. Ethics and Dissemination

This trial will be registered in the WHO International Clinical Trials Registry Platform (ICTRP) http://www.chiati.org.cn/index.aspx, accessed on 4 January 2021). A written informed consent will be obtained from each participant. Ethical approval will be obtained from the Ethics Committees of all involved institutions. All procedures performed in this study will be in accordance with the ethical standards of the institutional research committee and with the Helsinki Declaration and its later amendments. Any changes that need to be made in the trial protocol will be communicated to all investigators, the ethics committees, and the trial registry. The results obtained will be disseminated in peer-reviewed journals and presented at international conferences.

## 4. Discussion

### 4.1. Limitation of Previous Trials

As we mentioned before, from a previous revision of the literature, we concluded that for an accurate assessment of acupuncture as a valuable tool in PCOS syndrome treatment, more blinded RTC studies with larger samples are necessary. Moreover, we think that future studies should include adequate controls and the study designs should follow STRICTA recommendations. To make it possible for interstudy comparison, we think that it would also be important that the PCOS diagnostic criteria should meet the latest Rotterdam criteria with the discrimination of the types of PCOS phenotypes [48] since the severity of the disease decreases from A to D phenotype [28]. With respect to outcomes, our previous review of the literature revealed that most of the studies mainly focused on the ovarian dysfunctions such as infertility, hormonal ratio LH/FSH, menstrual frequency, and ovulatory and pregnancy rate values [53,54,55,56]. On the other hand, hormonal studies contemplated levels of FSH and LH, but no other important biomarkers that, in our opinion, would support better assessment of biochemical hyperandrogenism and hypothalamus–pituitary–gonad/adrenal (HPG/A) dysregulation, such as androstenedione, dehydroepiandrosterone and sex-hormone-binding globulin (SHBG). Additionally, the evidence for long-term efficacy of acupuncture in patients with PCOS is yet insufficient. In summary, we think that acupuncture studies on PCOS should use a broader analysis of sex hormones and assess secondary outcomes to describe the metabolic status of each phenotype, before and after treatment, and have a follow-up analysis. Furthermore, we think it would be important to use international measures to facilitate interstudy comparability.

### 4.2. Proposed Trial

To overcome the described limitations found in the literature on PCOS women studies, we propose an RCT with a sample of 120 women. This large sample would be important to increase the probability of including participants from all 4 phenotypes of latest Rotterdam criteria. Additionally, we will include a control group. Not all the previous studies presented blank controls [54,56] which, in our opinion, will be fundamental to understanding the effect of acupuncture on the PCOS condition. The design of our study follows the SPIRIT checklist and the international guidelines recommended for every released RCT [57]. Our study design will use the traditional Chinese medicine diagnostic with the respective rationale for the general condition of PCOS and the specific symptoms or signs. Thus, patients will be divided into two main phenotypes according to the TCM pathomechanism proposed by Lyttleton (2013). Although reproductive cycle normalization is a common indicator of a healthy reproductive status, we think it is more accurate to use the increase of the ovulation rate, balance of sex hormones, and improvement of the metabolic profile as the main indicators of the efficiency of the trial. For this reason, we suggest a study where the analysis of the results is divided in three parts: primary, secondary, and exploratory outcomes.

### 4.3. What We Expect with the Trial

With the proposed trial, we expect to validate an acupuncture protocol in terms of efficacy and safety that can be a useful tool in the management of symptoms associated with PCOS. With this study design, we expect to find evidence that active acupuncture can be more helpful for promoting follicular development, adjusting the menstrual cycle, and increasing ovulation rate in women with PCOS than sham acupuncture and the long-term effect of the treatment. After active acupuncture treatment, it is expected to find a relative reduction of the biomarkers when comparing to baseline values regarding LH, LH/FSH, total free testosterone, androstenedione, estradiol, BMI, waist circumference, WRH, modified Ferriman–Gallwey score, and Ludwig visual score. We expect an improvement of biochemical hyperandrogenism and HPG axis imbalance, a decrease of androstenedione and sex-hormone-binding globulin, besides the expected decrease in the levels of LH, LH/FSH ratio, total testosterone, estradiol, and on the scoring of the modified Ferriman–Gallwey and Ludwig scales. We expect a possible increase in FSH, progesterone, and the ovulation rate after active acupuncture. With respect to secondary outcomes, we will explore the effect of the acupuncture treatment on metabolic symptoms and expect to describe the metabolic profile of each phenotype before and after the respective treatment. We will adopt international measures as a gold standard for assessing the metabolic status criteria. Besides HOMA-IR improvement, we expect to find a positive correlation between active acupuncture and reduction of the levels of triglycerides, total cholesterol, LDL-C, LDL/HDL, fasting glucose, fasting insulin, and serum insulin and an overview improvement of the metabolic syndrome development. In addition to that, we will try to understand the efficacy of the treatment on exploratory parameters related to the quality of life of the patient, many times disregarded in this type of treatment, especially in anxiety and depression scoring. In this regard, we expect to find a better scoring in the respective questionnaires (reduction in the scoring points of Zung Depression and Anxiety scales and a gain of points in the SF-36 questionnaire) after active acupuncture intervention, particularly in phenotypes where liver fire associated with kidney deficiency originates a lack of mental presence or easy loss of temper. With the results of the study, we also expect to obtain evidence on the possible correlation of phenotypes based on the Rotterdam criteria and those based on the TCM diagnosis proposed by Wang et al., 2017.

## 5. Conclusions

The expected results of the proposed RCT study could provide evidence of high methodological quality related to the effect and safety of an acupuncture protocol based on the perspective of a TCM diagnostic, with the evaluation of improvement in hormonal, metabolic, and emotional symptoms associated with PCOS.

## Figures and Tables

**Table 1:** Rotterdam phenotypes for PCOS women with correspondent TCM phenotype.

Phenotype	Description
Rotterdam Phenotype A (classical PCOS) [46]	Hyperandrogenism + polycystic ovary morphology + ovulatory dysfunction—28.7%
TCM phenotype [47]	Spleen deficiency and phlegm stasis SP’s—32.27%
Rotterdam Phenotype B [46]	Hyperandrogenism + Ovulatory disfunction—19.0%
TCM phenotype [47]	Phlegm stasis and blood stasis—16.55%
Rotterdam Phenotype C [46]	Ovulatory disfunction + polycystic ovary morphology—37.3%
TCM phenotype [47]	Kidney deficiency and liver stasis—40.29%
Rotterdam Phenotype D [46]	Hyperandrogenism + polycystic ovary morphology—15.0%
TCM phenotype [47]	Kidney deficiency and blood stasis—10.89%

**Table 2:** SPIRIT schedule of enrolment, interventions, and assessments.

	STUDY PERIOD														
	Enrolment	Allocation	Post-Allocation												Close-Out
TIMEPOINT	*−1 month*	0	*_W1_*	*_W2_*	*_W3_*	*_W4_*	*_W5_*	*_W6_*	*_W7_*	*_W8_*	*_W9_*	*_W10_*	*_W11_*	*_W12_*	*After 6 months*
ENROLMENT:															
Eligibility screen	X														
Informed consent	X														
Baseline assessments	X														
Allocation		X													
INTERVENTIONS:															
Active acupuncture			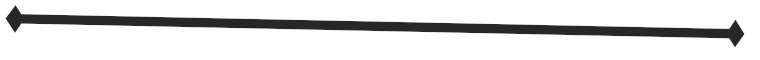												
		X	X	X	X		X		X		X		X	
Sham acupuncture			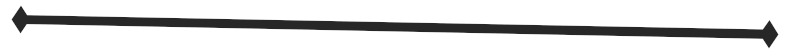												
		X	X	X	X		X		X		X		X	
ASSESSMENTS:															
*Diagnostic examination*	X														
*Primary and secondary outcomes*		X						X						X	X
*Life quality related questionnaires*	X	X						X						X	X

**Table 3:** Primary, secondary, and exploratory outcomes that will be used in the study.

Primary	Secondary	Exploratory
**Hormonal Parameters**: Modified Ferriman-Gallwey score Ludwig visual score LH, mean (SD), mIU/mL FSH, mean (SD), mIU/mL LH/FSH Total free testosterone Androstenedione Progesterone, median (IQR), ng/mL Estradiol, median (IQR), pg/mL Sex hormone–binding globulin (SHBG), mean (SD), μg/mL Ovulation rate (%)	**Anthropometric parameters** Age (years) Height (cm) Weight (Kg) BMI (kg/m^2^) Waist circumference (cm) Waist Hip Ratio	**Quality-of-life scores**: Medical Outcomes Study 36-Item Short Form Health Survey Zung Self-Rating Anxiety Scale Zung Self-Rating Depression
	**Metabolic parameters** TG (mg/dL) Total cholesterol, mean (SD), mg/dL LDL/HDL LDL-C (mg/dL) HDL-C(mg/dL) HOMA-IR Fasting glucose (mg/dL) Fasting insulin, mean (SD), μIU/mL Serum insulin (mU/L) Metabolic syndrome, No/total (%)	

## Appendix A

**Table A1:** Description of the active and sham acupuncture protocol proposal for women with PCOS based on a recent literature review.

Active Acupuncture			
Group I–Kidney Yang Deficiency with Phlegm-Dampness			
Acupoint	Location	Muscle Innervation	TCM Action
REN-3 (Zhōngjí)	4 cun below the umbilicus on the mid-line	L1	Resolve dampness on genital system
REN-4 (Gǔangyúan)	On the midline, 3 cun inferior to the umbilicus	Th12	Tonify kidney yang
Ren-12 (Zhōngwăn)	4 cun above centre of umbilicus	Th7-Th8	Harmonize and strengthen stomach, spleen and the Middle Jiao vessels; Fortify Shen
Bilateral SP-6 (Sānyīnjiāo)	On the medial aspect of the lower leg, 3 cun above the medial malleolus, on the posterior border of the medial aspect of the tibia.	L4-L5; S1-S2	Resolve dampness
Bilateral ST-25 (Tiānshū)	2 cun lateral to center of umbilicus	Th6-Th12	Remove Qi and blood stasis; harmonize spleen and stomach; resolve damp and damp-heat
Bilateral LIV-3 (Tàichōng)	Just distal to the junction of the bases of the 1st and 2nd metatarsals	S2-S3	Resolve damp-heat in the lower burner; sooth the liver; direct Qi in the connecting vessels and quicken the blood.
Bilateral KID-3 (Tàixī)	In the depression between the medial malleolus and the Achilles tendon, level with the tip of the medial malleolus	L4	Tonify kidney Yang
DU-20 (Bàihuì)	On the midline of the head, 7 cun directly above the posterior hairline, approximately on the midpoint of the line connecting the apexes of the two auricles.	C2-C3; Trigeminus nerve	Clears the mind, lifts the spirits, tonifies Yang, strengthens the ascending function of the spleen, eliminates interior wind
Right BL-62 (Shēnmài) coupled to Left SI-3 (Hòuxī)	Bl 62: On the lateral side of the foot, approximately 0.5 cun inferior to the inferior border of the lateral malleolus, in a depression posterior to the peroneal tendons. Si3: On the ulnar border of the hand, in the substantial depression proximal to the head of the fifth metacarpal bone.	S1 coupled to C8	Clears the spirit-disposition; dispels interior heat; frees the governing vessel
**Group II–Kidney Yang Deficiency with Blood Stasis**			
REN-3 (Zhōngjí)	4 cun below the umbilicus on the mid-line	L1	Resolve dampness on genital system
REN-4 (Gǔangyúan)	On the midline, 3 cun inferior to the umbilicus	Th12	Tonify kidney Yang
REN-6 (Qìhăi)	4 cun above centre of umbilicus	Th7-Th8	Harmonize and strengthen stomach, spleen and the Middle Jiao vessels; fortify Shen
Bilateral SP-9 (Yīlíngqúan)	Under the medial condyle of the tibia, in the depression posterior and inferior to the medial border of the tibia.	S1-S2	Resolve dampness
Bilateral ST-25 (Tiānshū)	2 cun lateral to center of umbilicus	Th6-Th12	Remove Qi and blood stasis; harmonize spleen and stomach; resolve damp and damp-heat
Bilateral ST-29 (Guīlái)	On the lower abdomen, 4 cun below the center of the umbilicus, 2 cun lateral to the anterior median line.	Th6-Th12	Invigorate blood and eliminate stasis
Bilateral KID-3 (Tàixī)	In the depression between the medial malleolus and the Achilles tendon, level with the tip of the medial malleolus	L4	Tonify kidney Yang
Left KID-6 (Zhàohăi) coupled with Right LU-7 (Lièquè)	K6:1 cun inferior to the tip of the medial malleolus, in a depression formed by two ligamentous bundles Lu 7: 1.5 cun proximal to the most distal skin crease of the wrist, proximal to the styloid of the radius in a depression between the tendons of brachioradialis and abductor pollicis longus.	S1 coupled with C7	Regulate the directing vessel and strengthen the uterus; help to dissolve the masses
DU-20 (Bàihuì)	On the midline of the head, 7 cun directly above the posterior hairline, approximately on the midpoint of the line connecting the apexes of the two auricles.	C2-C3; Trigeminus nerve	Clears the mind, lifts the spirits, tonifies Yang, strengthens the ascending function of the spleen, eliminates interior wind
P-6 (Nèiguān) coupled to Left SP-4 (Gōngsūn)	P6: on the palmar aspect of the forearm, 2 cun above the transverse crease of the wrist, on the line connecting PC 3 and PC 7, between the tendons of m. palmaris longus and m. flexor carpi radialis. Sp4: just distal and inferior to the proximal end of the metatarsal bone of the big toe	C8-Th1 coupled to L4?	Nourish blood in Chong Mai and strengthen the uterus
**Sham Acupuncture**			
**Acupoint**	**Location**	**Skin Innervation**	**TCM Action**
No known point	On top of acromion	C3-4, n. Supraclavicularis	No known action
No known point	On umerus lateral side of the upper arm	C5-6, n. cutaneous brachiilateralis	No known action
